# Post stroke fall and associated factors among stroke survivors at hospitals in Jimma town, oromia regional state, Southwest Ethiopia, 2025: a cross sectional study

**DOI:** 10.1186/s12883-025-04320-3

**Published:** 2025-08-09

**Authors:** Yohannes Girma Legese, Foad Akmel, Keiru Abajebal, Dechasa Imiru Wayessa, Sisay Deme, Yazachew Mekonnen, Tesfalem Yitbarek

**Affiliations:** Department of Physiotherapy, Faculty of Medical Sciences, Institute of Health, Jimma University, Jimma, P.O. Box: 378, Ethiopia

**Keywords:** Post-Stroke fall, Stroke, Cerebrovascular accident, Ethiopia

## Abstract

**Background:**

Post-stroke fall is a common incidence among stroke survivors, and it has several detrimental effects on this group of people. It significantly influences their well-being, increasing morbidity and reducing functional independence. It also leads to limiting activity and participation, increasing dependence and developing a fear of movements. In addition, it delay the progress of motor and cognitive recovery, as the patients may avoid activities that increase their risk of falling. Therefore, assessing post-stroke falls and their associated factors is necessary to address their consequences.

**Subjects:**

Stroke survivors who attended stroke units and physiotherapy outpatient clinics at hospitals in Jimma town, Oromia Regional State, South west Ethiopia.

**Method:**

A hospital-based cross-sectional study with a systematic random sampling technique was employed, and the data collection occurred through chart review, physical examination, and face-to-face interviews. The collected data was analyzed on SPSS Version 25. Bi-variable analysis was used to determine potential candidate variables. Finally, an adjusted odds ratio with a *P* value < 0.05 and a 95% confidence interval was considered statistically significant.

**Result:**

Among the study participants, 59.5% (235) of stroke survivors experienced post-stroke falls. Key factors includes an older age group (AOR = 3.2; 95% CI = 1.159**–**9.020), hemorrhagic type of stroke (AOR = 1.8; 95% CI = 1.036**–**3.088), spastic muscle tone (AOR = 2.7; 95% CI = 1.343**–**5.394), altered mental status (AOR = 1.7; 95% CI = 1.019**–**2.819), less number one caregiver (AOR = 1.8; 95% CI = 1.119**–**3.026), and late admission to hospital (AOR = 2.1; 95% CI = 1.115–3.952).

**Conclusion:**

More than half of stroke survivors had a history Post-stroke falls in the Jimma town with key factors including older age, type of stroke, spastic muscle tone, ≤one caregiver, late admission to hospital, and altered mental status.

**Supplementary Information:**

The online version contains supplementary material available at 10.1186/s12883-025-04320-3.

## Background

A stroke/cerebrovascular accident occurs when blood flow to the brain is disrupted, either by a blockage or by a burst blood vessel, leading to brain damage. It remains the second leading cause of death worldwide and a major cause of disability worldwide, especially in low- and middle-income countries [1]. The prevalence and mortality rate of stroke are increasing globally, with the majority of the burden falling on low- and middle-income countries, including Ethiopia. In Ethiopia the pooled burden of hemorrhagic and ischemic stroke were 46.42% and 51.40% respectively [2]. The magnitude of stroke-related deaths is 6.23%, and the age-adjusted death rate of stroke is 89.82 per 100,000 of the population. The overall in-hospital mortality of stroke in Ethiopia was 15.1% before 2016 and increased to 18% in 2020 [3]. Furthermore, the proportion of stroke recovery was decreased after 2017 in the country, which reflects the increased exposure of stroke and stroke patients’ suboptimal care regimen, including lack of rehabilitation in the country [2].

Following a stroke, numerous complications comprising physical, psychological, and physiological problems might arise. Post-stroke fall (PSF) is among the most common complications that happen to patients with stroke. An estimated 14–65% of stroke patients will fall at least once while they are in the hospital. The rate of falls among stroke survivors in the first six months after leaving the hospital is between 37% and 73%, and the rate of repeat falls among stroke survivors up to a year after the stroke stays high at 20–57% [4]. 3% of stroke survivors fell in African nations like Nigeria [5].

In stroke rehabilitation, minimizing falls after a stroke is crucial since falls increase morbidity and decrease functional independence in these individuals. Such that those who have had a PSF are far more likely to experience limiting activity and participation, increasing dependence and developing a fear of movements [6]. Furthermore, stroke patients are at a heightened risk of falls not just during the acute phase but also throughout their post-stroke lives, which presents a serious health risk [7].

The high prevalence of falls across various cultural and geographic contexts was found in a multi-study comprising stroke survivors from Europe, Asia, and Australia, underlining the issue’s universality across stroke survivor [8]. Several factors affecting the risk of PSF among stroke survivors include socio-demographic factors, motor impairments, and stroke duration, with comorbidity and depressive symptoms identified as significant contributors [9, 10]. Therefore, identifying precise factors of PSF in our nation is crucial for preventing falls and injuries among stroke survivors. Although the proportion of stroke recovery was decreased, and PSF is a main concern that delays recovery, to our knowledge, there are no published studies regarding the prevalence and associated factors of PSF among stroke survivors in the study area and in our country. Therefore, this study aimed to assess the prevalence and associated factors of post stroke fall among stroke survivors at Hospitals in Jimma Town, Southwest Ethiopia.

### Objectives of the study

To assess the prevalence of post stroke fall among stroke survivors at Hospitals in Jimma Town, Southwest Ethiopia.

To assess associated factors of post stroke fall among stroke survivors at Hospitals in Jimma Town, Southwest Ethiopia.

## Method and material

### Study design and period

An institutional-based cross-sectional study design was conducted from December 2024 to March 2025.

### Study area and setting

An institutional-based cross-sectional study was conducted at hospitals in Jimma, Oromia Regional State, South west Ethiopia. Jimma town is one of the largest towns in the Oromia Regional State, located 346 km southwest of the capital city of Ethiopia, Addis Ababa, with a total population of 184,925. The town is geographically located at a latitude and longitude of 7°40′N, 36°50′E [11]. In Jimma town, there are two public hospitals (Jimma University Medical Center (JUMC) and Shanen Gibe General Hospitals (SGGH)) and four private hospitals (Oda Hullee Hospital (OHH), Hawetu Hospital (HH), Green Valley Hospital (GVH) and Firomsis Hospital (FH)).

### Eligibility criteria

All stroke survivors aged ≥ 18 who attended stroke unit and physiotherapy outpatient clinics at hospitals in Jimma, Oromia Regional State, southwest Ethiopia, during the study period were included. Participants with post stroke barthel Index score ≤ 10 did not participate in the study.

### Sample size determination

The single population proportion formula was used to calculate the sample size under the following presumptions. The prevalence of PSF among patients was assumed 50% to ensure the study was adequately powered, due to no previous study having been found locally (in the study area and in the country). By assuming a 5% margin of error (d), and a 95% confidence level (alpha, α = 0.05). Where: *P* = 50% due to the lack of a similar study in the study area, so to obtain the maximum sample size, P was taken as 50%), Zα/2 = critical value of the Z score at a 95% confidence interval of certainty, d = margin of sampling error tolerated (desired precision), 5% (0.05), $\:\text{n}=\frac{{\left(Z{\upalpha\:}/2\right)}^{2}\text{P}\:(1-\text{P})}{{\text{d}}^{2}}$ ≈ 385. By considering a 10% non-response rate, the final sample size was ≈ 424.

For second objective; by the study conducted in Serbia to identify risk factors of fall among stroke survival, in which mental status were recognized as one of the more important factor for falling after stroke [12]. The study had 235 were oriented and 326 were un-oriented participants. From 235 who were oriented, 81(34.5%) have fall, and from un-oriented (326), 171 (52.5%) exposed to fall. By assuming a 5% margin of error (d), 95% confidence level (alpha, α = 0.05, two-tailed), and 80% power (1−𝛽) to detect the assumed difference. This is calculated using epi info version 7, Z = confidence interval at 95%=1.96 the sample will be 266, by adding a 10% non-response rate, the sample will be 293. Since the sample size of the first objective is the largest, the final sample size for this study was 424.

### Sampling technique and procedure

Those six hospitals located in jimma town were included in the study. Based on the estimated monthly reports for four months, the total number of stroke survivors for was 765; with an estimated flow were 240, 120, 100, 110, 80 and 115 stroke survivors in JUMC, SGGH, OHH, HH, GVH and FH respectively.

The sampling interval, K^th,^ was calculated by dividing the total number of study participants at the study period by the study sample (N/n), which was 765/424 ≈ 2. To determine study participants for each selected hospital, the total sample size was proportionally allocated for each hospital using the statistical proportional allocation formula (ni = n*Ni/N), where n is the total sample size to be selected, N is the total number of patients, Ni is the number of patients in each hospital, and ni is the sample size for each hospital cardiac unit. Based on the above proportional allocation, the study participants for each selected hospital were 133, 67, 55, 61, 44 and 64 stroke survivors in JUMC, SGGH, OHH, HH, GVH and FH respectively. So that each selected hospitals, k value was approximately 2. Then the first patient in each hospitals were selected using the lottery method between one and “k” = 1–2, and it was 1, and other patients were selected by the systematic random sampling method within every 2nd value until the required sample size was obtained when patients attends each hospital.

### Variable of the study

#### Dependent variable

Post-stroke fall (Yes/No).

#### Independent variable

##### Socio-demographic factors

Age, Gender, Marital status, BMI, Level of education, occupation, Residence, Household income.

##### Health-related factors

Time since onset, type of stroke, side of the brain affected, comorbidity, altered mental status, tone Abnormality, time to hospital admission.

##### Personal and environmental factors

Smoking, alcohol consumption, and number of caregivers.

### Operational definition

#### Post-stroke fall

any fall regardless of cause that could be identified by staff/patient/caregiver or documented in patients’ medical record/hospital notes [13].

#### Body mass index (BMI)

measured by dividing weight in kilograms by the square of height in meters (kg/m2). Individuals are considered underweight (< 18.5), normal (18.5–24.9), overweight (≥ 25.00-29.9), and obese (30.0 and above) [14].

#### Current smoker

someone who has smoked greater than 100 cigarettes in their lifetime and who now smokes every day [15].

#### Spasticity

based on Modified Ashworth Scale the cutoff point for defining clinically significant spasticity is a score of 1 or higher [16].

#### Comorbidity

coexistence of chronic health conditions with the present cardiac disease; includes systemic, physical, or mental health conditions [17].

#### Altered mental status

A score of 23 or lower is indicative of altered mental status based on Mini Mental State Examination [18].

### Data collection tool, procedure and data quality assurance

Data were collected using interviews method, patient medical chart reviews and examination to collect the necessary data. A semi-structured questionnaire was developed for this study using a literature review [13, 19–24] and used for recording socio-demographic, health-related factors, contextual factors, and fall-related characteristics. The questionnaire had four parts. Part one of the questionnaire focuses on the different socio-demographic factors of the patients. In part two of the questionnaire health-related conditions such as type of stroke, comorbidity, affected side of the brain, duration of stroke, duration to hospital admission, muscle tone and mental status were recorded from patients medical charts and examination, which is the most recent one [19, 25]. In part three of the questionnaire, personal and environmental factors related questionnaires like alcohol intake, smoking habits and number of caregivers were recorded using self-reported questions. Part four of the questionnaire consisted of question on the history of falls and injury related to fall [13, 22]. (Appendix 1)

The quality of the data was controlled starting at the time of questionnaire preparation. To ensure the quality of the data, the questionnaire was first prepared in English, then translated into the local languages Amharic and Oromic, and then back to English to maintain its consistency. The data collectors were six trained physiotherapy students. To maintain the quality of the data, all data collectors were trained for one day on how to approach and collect the data on study participants, how to use the questionnaire, and how to get informed consent from the study participants. Before the actual data collection time, the tool was pretested to check for the accuracy of responses, language clarity, consistency, and appropriateness of the tools with 5% of the total sample size (22 patients).

Data collection was started by screening stroke patients who volunteered to participate in the study based on the inclusion and exclusion criteria. After the importance of the study was explained and informed consent was obtained, data collectors collected the data from participants using the translated version of the questionnaire.

### Data processing and analysis

Using Epi Info version 7, the gathered data was coded, cleaned, and entered. Once exported, SPSS version 25 was used for data analysis. Descriptive statistics were done for all the variables in the study using statistical measurements including frequency tables, and percentages. The binary logistic regression was employed to show the relationship between dependent and independent variables. Variables with a p-value less than 0.25 in the bi-variable analysis were further analyzed using a multi variable logistic regression model to control for confounding factors. The interpretation was made using the adjusted odds ratio (AOR) with its corresponding 95% confidence interval. Variables having a p-value less < than 0.05 in the multivariable logistic analysis were taken as a significant factor for PSF. The model fitness was checked using Hosmer and Lemeshow’s goodness of fit test, which shows that the model is fit(0.626), and the multicollinearity diagnostic was performed by the variance inflation factor (VIF < 10).

## Result

### Socio-demographic characteristics of the respondents

A total of 395 participants were included in the study, making the response rate 93%. The majority of participants, 49.1%, were in the age of 40–60 years, and most of the respondents were female, 207 (52.4%), and 257 (65.1%) were currently married. More than one-fourth of the study participants had no formal education: 115 (29.1%). (Table 1).

**Table 1:** Socio-demographic related characteristics among stroke survivors at hospitals, in Jimma town, ethiopia, 2025 (*n* = 395)

Variables		Frequency (*n*)	Percent (%)
Age	20–39	38	9.6
	0–59	194	49.1
	≥ 60	163	41.3
Sex	Female	207	52.4
	Male	188	47.6
Level of education	No formal education	115	29.1
	Primary education	99	25.1
	secondary education	98	24.8
	College and above	83	21.0
Marital status	Single	45	11.4
	Married	257	65.1
	Divorced/Widowed	93	23.5
Occupation	Gov’t employee	54	13.7
	Non-Gov’t employee	195	49.3
	unemployed	146	37
Residence	Urban	243	61.5
	Rural	152	38.5
Income (ETB)	< 2000	49	12.4
	2000–5000	92	23.3
	> 5000	254	64.3
BMI (kg/m^2^)	< 18.5	154	39.0
	18.5–24.9	147	37.2
	≥ 25	94	23.8

_ETB−Ethiopian Birr, BMI−Body Mass Index, Gov’t−Governmental_

### Health-related and clinical characteristics of stroke survival

Of the total respondents, 121 (30.6%) had chronic stroke. Among the total participants, 205 (51.9%) of the respondents had spastic tone, and 96 (24.3%) of the respondents had at least one comorbid condition. Further, in 59% of participants, the right side of the brain was affected and 75.9% and 56.7% had been admitted to the hospital within 12 h of stroke onset and had an altered mental status, respectively. (Table 2).

**Table 2:** Description of health related characteristics among stroke survivors at hospitals, in Jimma town, ethiopia, 2025 (*n* = 395)

Variables		Frequency(*n*)	Percent (%)
Types of stroke	Ischemic	293	74.2
	Hemorrhagic	102	25.8
Comorbidity	Yes	96	24.3
	No	299	75.7
The side of the brain affected	Right side	233	59.0
	Left side	162	41.0
Muscle tone	Normal	60	15.2
	Flaccid	130	32.9
	spastic muscle tone	205	51.9
Duration of onset	< 3 months	124	31.4
	3–6 months	150	38.0
	>6 months	121	30.6
Admission to hospital	< 12 h.	300	75.9
	≥ 12 h.	95	24.1
Altered mental status	Altered	224	56.7
	Normal	171	43.3

### Lifestyle-related and environmental factors of study participants

Among the study participants, 374 (94.7%) were non-alcoholic; 373 (94.4%) were not current smokers. Regarding environmental-related characteristics, more than two thirds of the study participants, 278 (70.4%), had less than one caregiver. (Table 3).

**Table 3:** Lifestyle and environmental factors among stroke survivors at hospitals, in Jimma town, ethiopia, 2025 (*n* = 395)

	Variable	Frequency	Percent
Alcohol habit	No	374	94.7
	Yes	21	5.3
Current smoking	No	373	94.4
	Yes	22	5.6
Number of caregivers	≤ 1 caregiver	278	70.4
	≥ 2 caregivers	117	29.6

### Prevalence of fall among stroke survivals

The current study finding revealed that the prevalence of falls among stroke survivors who visited hospitals in Jimma town was 59.5% (235) (95% CI; 54.5, 64.4).

Among a total of 235 study participants, those who had a history of falling, most of the study participants, 196 (83.4%), had a number of falls less than two times. In addition, most study participants, 207 (88.1%), fell during daytime, and more than two thirds of the study participants, 171 (72.8%), had indoor falls. (Table 4).

**Table 4:** Description of post-stroke fall and related characteristics of stroke survivors at hospitals, in Jimma town, ethiopia, 2025 (*n* = 235)

Variables		Frequency(*n*)	Percent (%)
Number of falls	≤ 2	196	83.4
	> 2	39	16.6
Location of fall occurrence	Outdoor	64	27.2
	Indoor	171	72.8
When fall occur	day time	207	88.1
	night time	28	11.9

From those who had a history of falling (235), 63.4% sustained some type of injury. Regarding the affected body part after PSF, the upper extremity was the most affected region, accounting for 33.5%, followed by lower extremity 20.1%, back 14%, head injury 2.5%. While 21.4% of the participants had multiple injuries and 8% of participants have, unknown body part affected. (Table 5).

**Table 5:** Consequence of post stroke fall among stroke survivals at hospitals, in Jimma town, ethiopia, 2025 (*n* = 235)

Variables		Frequency(*n*)	Percent (%)
Injury after fall	Yes	149	63.4
	No	86	36.6
Nature of injury	soft tissue injury	105	70.5
	Fracture	11	7.4
	undefined injury	33	22.1

### Factors associated with post-stroke fall

In bivariate analysis, age, gender, residency, BMI, type of stroke, muscle tone, altered mental status, number of caregivers, duration of the disease, time to hospital admission, and comorbidity showed significant associations with PSF.

In multivariable logistic analysis, factors that showed significant associations were an older age group (AOR = 3.2; 95% CI = 1.159**–**9.020), hemorrhagic type of stroke (AOR = 1.8; 95% CI = 1.036**–**3.088), spastic muscle tone (AOR = 2.7; 95% CI = 1.343**–**5.394), altered mental status (AOR = 1.7; 95% CI = 1.019**–**2.819), less number one caregiver (AOR = 1.8; 95% CI = 1.119**–**3.026), and late admission to hospital (AOR = 2.1; 95% CI = 1.115–3.952) with a p-value less than 0.05. (Table 6).

**Table 6:** Factors associated with post-stroke fall among stroke survivors at hospitals, in Jimma town, ethiopia, 2025 (*n* = 395)

Variables	PSF		Multi variable	
	No	Yes	AOR(95%CI)	*P*-value
Sex				
Female	78	129	1	
Male	82	106	1.212 (0.751-1.956)	0.430
Age				
< 39	18	20	1	
40–59	98	96	0.902 (0.342-2.375)	0.834
> 60	44	119	3.234 **(**1.159**–**9.020) *	0.025
Residence				
Rural	51	101	1.526 (0.938-2.482)	0.089
Urban	109	134	1	
BMI				
18.5–24.9	67	80	1	
< 18.5	63	91	1.117 (0.663-1.882)	0.678
≥ 25	30	64	1.773 (0.928-3.385)	0.083
Type of stroke				
Ischemic	124	169	1	
Hemorrhagic	36	66	1.789 **(**1.036**–**3.088) *	0.037
Tone				
Normal	38	22	1	
Flaccid	68	62	1.119 (0.558-2.245)	0.752
Spasticity	54	151	2.692 **(**1.343**–**5.394**)** *	0.005
Altered mental status				
Altered	74	150	1.695 **(**1.019**–**2.819)*	0.042
Normal	86	85	1	
No. of caregivers				
≤one caregiver	96	182	1.840 **(**1.119**–**3.026**)** *	0.016
>one caregivers	64	53	1	
Admission to hospital				
< 12 h.	139	161	1	
> 12 h.	21	74	2.099 (1.115–3.952) *	0.022
Comorbidity				
Yes	34	62	1	
No	126	173	1.735 (0.977-3.082)	0.060
Duration of onset				
< 3 months	47	77	1	
3–6 months	77	73	1.034 (0.486-2.201)	0.930
>6 months	36	85	0.770 **(**0.330**-**1.797)	0.546

*COR* = crude odds ratio, *AOR* = Adjusted odds ratio, 1 = Reference, * statistically significant at a *p*-value < 0.05, *CI* = confidence interval

## Discussion

The purpose of this study was to determine the magnitude of PSF and its associated factors among stroke survivors at hospitals in Jimma town. The current study found that the prevalence of PSF at hospitals in Jimma town was 59.5%% and being old, having a hemorrhagic type of stroke, having spastic muscle tone, having altered mental status, having a longer time to hospital admission, and having a smaller number of caregivers were significantly associated with PSF.

However, the prevalence of our study was higher than the previous study in Sweden and New Zealand that reported 8.5% and 39% prevalence of PSF [26, 27]. The discrepancy might be due to patients in those countries having better access to standard care and rehabilitation programs. On the contrary, the prevalence of this study was lower than studies done in the UK, Bangladesh, and Serbia among stroke survivors [12, 13, 28]. This discrepancy might be due to differences in methodology, study setting, and socio-demographic variation.

According to our study, participants whose age was significantly associated with PSF. Our study implies that participants who were age > 60 were 3.2 times more likely to fall after stroke as compared to those with ages below 40. The relationship between PSF and age also supported by the studies done in Nigeria, Korea and Sweden [5, 29, 30]. As individuals age, they may naturally experience declines in muscle strength, balance, and coordination [31], these age-related changes can be exacerbated by the effects of a stroke, making older adults more vulnerable to falls.

This study showed that type of stroke was significantly associated with PSF; having a hemorrhagic stroke was 1.8 times more likely to have PSF. This association was in line with a study done in Sweden [26]. This may be hemorrhagic strokes can cause significant neurological deficits, including weakness, paralysis, or loss of coordination on one side of the body [32]. These impairments can make it more difficult for patients to walk or move safely that increase the risk for fall.

Our study showed that stroke survivors who had spasticity were 2.7 times more likely to experience PSF than those who had no spasticity. Studies done in Taiwan and Turkey also aligned with this study finding [33, 34]. This might be spasticity can lead to difficulty in controlling movements and can make it hard for individuals to adjust their posture [35], increasing the likelihood of falling.

This study found that participants with altered mental status were 1.7 times more likely to experience PSF. The relationship between mental status and fall also supported by cross-sectional and retrospective research conducted in USA [36, 37]. Altered mental status results in decreased awareness of surroundings, making it difficult for stroke patients to take quick reactions and making it difficult for stroke patients to recognize potential hazards such as uneven floors, stairs, or obstacles in their path [38].

Our study showed that the number of caregivers was statistically significant with PSF. It was found that participants with less than one caregiver were 1.8 times more likely to experience PSF than those who had more caregivers. A follow up study done in Sweden also suggests stroke survivors who live alone were more likely to have PSF [29]. When the number of caregivers is reduced, the ones who are there are frequently overburdened and under stress. Reduced attentiveness, poor judgment, and an inability to adequately support the stroke patient are all consequences of caregiver burnout, which raises the risk of falls among stroke survivors [39].

Concerning the time of hospital admission, our study found that those patients who took > 12 h. to reach the hospital were 2.1 times more likely to have PSF compared to those who admitted to the hospital before 12 h. This might be due to early stroke management being important for stroke survivors, and patients who did not get early management are more likely to experience more severe and long-lasting neurological impairments, additional complications, and a delayed commencement of rehabilitation. Their risk of falling is greatly increased by these circumstances as compared to individuals who receive timely care and treatment [40, 41].

### Limitations of the study

This study addresses a significant health issue influencing stroke survivors by focusing on specific challenges and concerns that stroke survivors face. Despite this, a few limitations can be mentioned to benefit future researchers and to interpret this result with caution. Since the study employs a cross-sectional design, it may not adequately establish causal relationships between risk factors and PSF. Secondly, some variables like balance and other stroke-related impairments, such as visual impairment, were not assessed. In addition, this study was carried out only in a hospital environment at a point and did not address stroke survivors in the community. However, for future studies, it is recommended to address stroke survivors in the community.

## Conclusion and recommendation

This study found more than half of stroke survivors had a history of PSF at hospitals in Jimma town. Key factors significantly associated with PSF included old age, hemorrhagic type of stroke, spastic muscle tone, altered mental status, longer time to hospital admission, and number of caregivers. Addressing these risk factors during hospital care and in the general population can better support stroke survivors. Based on the identified associated factors, targeted fall-prevention interventions should be integrated into post-stroke rehabilitation programs. These findings highlight the need for multidisciplinary approaches in stroke aftercare, involving physiotherapists, occupational therapists, and primary care providers to address fall. Furthermore, by using this data, community-based rehabilitation programs and public health campaigns can prioritize high-risk populations and allocate resources effectively. Health professionals, including physiotherapists should give attention to risk factors of PSF during hospital care and in the community setting.

## Supplementary Information

Supplementary Material 1.

## Data Availability

The study contains all of the study data related to these findings. The data sets used and/or analyzed during the current study are available from the corresponding author on reasonable formal request.

## Abbreviations

AOR: Adjusted Odds Ratio
BMI: Body Mass Index
CI: Confidence Interval
COR: Crude Odds Ratio
PSF: Post Stroke Fall
SPSS: Statistical Package for the Social Sciences
