# Supplementary material for: Post stroke fall and associated factors among stroke survivors at hospitals in Jimma town, oromia regional state, Southwest Ethiopia, 2025: a cross sectional study
Source: BMC Neurol. 2025 Aug 9;25:329. doi: 10.1186/s12883-025-04320-3 (PMC12335792; doi:10.1186/s12883-025-04320-3)
Supplement: Supplementary file 1 — Supplementary Material 1. [file 12883_2025_4320_MOESM1_ESM.docx]

**Annex I: English version of Questionnaire: Self-reported post stroke fall among stroke survivors in Jimma town.**

**Questionnaire unique ID ___ ___ ___ ___ ___, Date of recording: __ __ __ __ __ Time: __ __**

**General instruction: Circle the choice among the alternatives; write a correct response on blank space for those questions which needs specification**

**Part 1: socio-demographic related factors of stroke survival**

| No | Question type | Response | Remark |
| --- | --- | --- | --- |
| SD1 | Sex | 1. Male 2. Female |  |
| SD2 | How old are you? | ---------- in years |  |
| SD3 | What is the highest level of your education completed? | 1. No formal education 2. Primary school 3. Secondary school 4. College and above |  |
| SD4 | What is your marital status? | 1. Single 2. Married 3. Separated 4. Divorced |  |
| SD5 | Occupation | 1. Governmental-employed, 2. Non-governmental employed 3. Unemployed |  |
| SD6 | Residence? | 1. Urban 2. Rural |  |
| SD7 | Can you tell me your monthly household  Income? | ----------birr(ETB) |  |
| SD8 | Weight? | ____________Kg |  |
| SD9 | Height? | ____ meter____ cm |  |

**Part 2: Health-related and clinical characteristics of stroke survival**

| No | Question type | Response | Remark |
| --- | --- | --- | --- |
| HC1 | Type of stroke? | 1. Hemorrhagic Stroke 2. Ischemic Stroke |  |
| HC2 | Duration of onset? | _______months |  |
| HC3 | Which side of the brain hemisphere is affected? | 1. Right hemisphere 2. Left hemisphere |  |
| HC4 | Patient status in terms of muscle tone? | 1. Normal muscle tone 2. Flaccid muscle tone 3. Spastic muscle tone |  |
| HC5 | Mental status? | 1. Altered 2. Normal |  |
| HC6 | Time from onset to hospitalization? | 1. ≤12Hr 2. ≥12Hr |  |
| HC7 | Any other medical problem? | 1. Yes 2. No |  |

**Part 3**: **Personal and Environmental factors**

| **Code** | Variables | Possible response |
| --- | --- | --- |
| PE1 | Number of Caregivers? | _____________ |
| PE2 | Do you smoke? |  |
| PE3 | If you say yes question (403/404), how many stick of cigarette you were smoked? | ………. |
| PE4 | Do you drink alcohols at some time in your life? | 1.Yes 2.No |

**Part 4.** **Assessing the history of falls**

| No | Question type | Response | Remark |
| --- | --- | --- | --- |
| F1 | Do you have any experience of falling after a stroke? | 1. Yes 2. No |  |
| F2 | If Yes, how many fall have you experienced? | _____ |  |
| F3 | Can you describe the location where the fall occurred? | 1. Indoor 2. Outdoor |  |
| F4 | What time of day did the fall happen? | 1. Daylight 2. Night time |  |
| F5 | Were you injured because of the fall(s)? | 1. Yes 2. No |  |
| F6 | If yes, please specify the nature of the injury/injuries | 1. Fracture 2. Soft tissue injury 3. Another injury |  |
| F7 | Where is the area of injury? | 1. head 2. upper extremity 3. back 4. lower extremity 5. multiple locations 6. unknown | 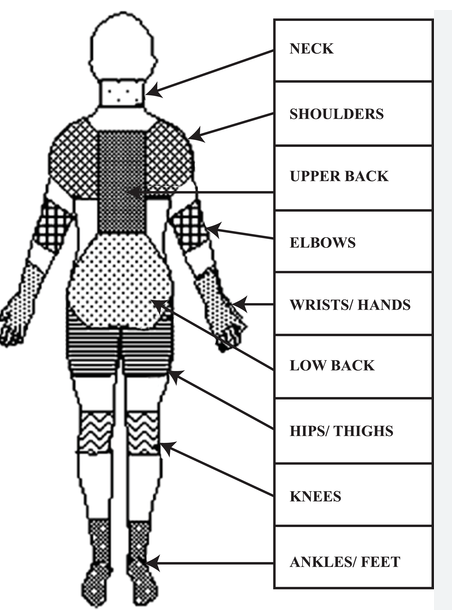 |
